# Blocking AMPK/ULK1-dependent autophagy promoted apoptosis and suppressed colon cancer growth

**DOI:** 10.1186/s12935-019-1054-0

**Published:** 2019-12-13

**Authors:** Jing Liu, Shuaiyu Long, Huanan Wang, Nannan Liu, Chuchu Zhang, Lingling Zhang, Yingjie Zhang

**Affiliations:** 1 College of Biology, Hunan University, Changsha, 410082 China; 2 Department of Laboratory Medicine, The Third Xiangya Hospital, Central South University, Changsha, 410013 China; 3 Department of Veterinary Medicine, College of Animal Sciences, Zhejiang University, Hangzhou, China; 4 Shenzhen Institute, Hunan University, Shenzhen, China

**Keywords:** Colon cancer, Autophagy, Apoptosis, NVP-BEZ235, CQ

## Abstract

**Background:**

Autophagy is an evolutionarily conserved process through which cells degrade and recycle cytoplasm. The relation among autophagy, apoptosis and tumor is highly controversial until now and the molecular mechanism is poorly understood.

**Methods:**

Cell viability and apoptosis were detected by CCK8, crystal violet staining, Hoechst333342 staining and flow cytometry. The expression of AMPK and ULK1 was analyzed by western blotting. Colon cancer growth suppression by NVP-BEZ235 or CQ in vivo was studied in a tumor xenograft mouse model.

**Results:**

Our previous study revealed that NVP-BEZ235 suppressed colorectal cancer growth via inducing apoptosis, however later, we found it also initiated autophagy simultaneously. In this present study, our results show that NVP-BEZ235 induced autophagy through AMPK/ULK1 pathway in colon cancer cells. Blocking autophagy by knocking down AMPK or ULK1 inhibited cell proliferation and further promoted NVP-BEZ235 induced apoptosis. Meantime, the autophagy inhibitor chloroquine (CQ) shows obvious effect on inhibiting cell proliferation but not on inducing apoptosis, while it significantly increased NVP-BEZ235 induced apoptosis. Furthermore, the combinational therapy of NVP-BEZ235 and CQ shows synergistic antitumor effects in colon cancer in vivo.

**Conclusion:**

NVP-BEZ235 induced AMPK/ULK1-dependent autophagy. Targeting this autophagy suppressed colon cancer growth through further promoting apoptosis, which is a potential therapeutic option for clinical patients.

## Background

Colorectal cancer is estimated about 6.1% incidence and 9.2% mortality in the world, the mortality rate is the second of the total cancer deaths in 2018 [1]. The cornerstones of therapy are surgery, however, for those patients in whom surgical resection is not possible, induction of apoptosis in tumor cells is a hopeful approach [2–4]. In our previous study, we have illustrated the mechanism that suppressed colon cancer growth through triggering apoptosis [5–7].

Autophagy is a process of self-destruction, cellular constituents including proteins and cytoplasmic organelles were orderly degraded and reusing [8, 9]. LC3 (microtubule-associated protein light chain 3) is now widely used to test autophagic activity, including LC3-I (cytosolic) and LC3-II (membrane bound). The amount of LC3-II is clearly associated with the number of autophagosomes, serving as a good indicator of the extent of autophagosome formation [10]. PI3K/AKT/mTOR signal pathway plays an important role in cell proliferation, survival and metabolism [11]. PI3K/Akt, and mTOR have been found to be over-activated in colorectal adenocarcinoma and have become potential targets for treatment [12, 13]. NVP-BEZ235, a dual PI3K/mTOR inhibitor, showing great therapeutic potential in colorectal adenocarcinoma and prostate cancer [7, 14]. Targeting PI3K/AKT/mTOR signaling can not only induce apoptosis to inhibit the proliferation of tumor cells, but also induce autophagy [15]. However, the crosstalk between autophagy and apoptosis was unclear [16, 17].

Autophagy initiation is regulated by Unc-51-like kinase 1 (ULK1) and there are two major upstream regulators: the mTOR complex 1 (mTORC1) and AMP-activated protein kinase (AMPK) [18, 19]. AMPK is an energy receptor, it regulates starvation-mediated autophagy induction, under nutrient sufficient, activation of AMPK by phosphorylation and promotion of pro-survival pathways [20]. Meantime, many studies have shown that AMPK activates autophagy by inhibiting mTORC1 [21]. Chloroquine (CQ), which blocks autophagy by impairing the fusion of autophagosomes with lysosomes and lysosomal protein degradation. In the past two decades, many publications have reported CQ combined with various of anticancer drugs to test medicines clinically effects [22–24].

Our previous study demonstrated that NVP-BEZ235 induced PUMA-dependent apoptosis suppressed colon cancer growth both in vitro and in vivo [7]. However, in this present study, we found that NVP-BEZ235 caused protective autophagy concurrently together with apoptosis in colon cancer. Further investigation illustrated that this autophagy is mediated by AMPK/ULK1 axis. So targeting autophagy by knocking down AMPK/ULK1 or by combinational treatment with CQ markedly enhanced the effect of NVP-BEZ235 on tumor growth suppression both in vitro and in vivo, which may provide a critical insight into colon cancer therapy.

## Materials and methods

### Cell culture and treatments

Human colorectal cancer cell lines (HCT116, SW48, RKO) were ordered from American Type Culture Collection (ATCC). HCT116 and SW48 were cultured in McCoy’s5A modified media (Invitrogen™) or DMEM medium (Gibco) routinely, RKO was cultured in Eagle’s minimum essential medium (EMEM), containing 10% fetal bovine serum (FBS), penicillin (100 units/mL), and streptomycin (100 mg/mL) in 37 °C incubator with 5% CO_2_ in humidified incubator. The agent of NVP-BEZ235 diluted with DMSO and CQ was dissolved in PBS. For the cell treatment, the concentrations of NVP-BEZ235 (400 nM), CQ (50 μM) or their combination mixed into the culture medium directly.

### Antibodies and reagents

Primary antibodies against ULK1, p-ULK1, AMPK, p-AMPK, LC3-II, cleaved-caspase3 and Actin were purchased from Cell Signaling Technology (CST). HRP-conjugated anti-rabbit or anti-mouse secondary antibodies and an ECL-plus kit were from Advansta in America. Chloroquine (CQ) was purchased from Sigma-Aldrich and NVP-BEZ235 was purchased from selleck. CCK-8 kit was from 7 sea biotech (Shanghai, China).

### Cell viability and apoptosis assays

HCT116, RKO and SW48 cells were been cultured in 96-well microplate at a density of 5 × 10^3^ cells/well for 24 h. Then, the cells were divided into several groups and treated with different conditions. Cell viability was assessed with CCK-8 at 24 h post-treatment according to the manufacturer’s instructions. The absorbance value at 450 nm (OD450) was read with a 96-well plate reader (DG5032, Hua dong, Nanjing, China), to determine the viability of the cells. For analysis of apoptosis by nuclear staining with Hoechst33342 (Invitrogen). Colon cells were cultured on the coverslip of a chamber, rinsed with phosphate-buffered saline (PBS) and then 500 mL DMEM/McCoy’s5A containing 5 μg Hoechst33342 was added in, incubated at 37 °C with 5% CO_2_ for 15 min. Apoptosis was being assessed through microscopic visualization of condensed chromatin and micro nucleation. For colony formation assays, equal number of cells after different treatments were planted in 6-well plates. Colonies were visualized by crystal violet staining 14 days after plating.

### Flow cytometry

For flow cytometry analysis (FACS analysis), human colon cancer cell lines with HCT116, RKO, SW48 were harvested in 1 × 10^5^ cells/mL after using different treatment. These groups were suspended by 100 μL binding buffer, and 5 μL Annexin V and 5 μL propidium iodide staining solution were added to the cell suspension respectively. Later, added 400 μL binding buffer into the cell suspension again. At room temperature, the cells were incubated in the dark for 10 min, and then cells were assayed and quantified using a FACSort Flow Cytometer (Beckman Coulter, Brea, CA, USA) at 488 nm. Fluorescent emission of FITC was measured at 515–545 nm and that of DNA-PI complexes at 564–606 nm. Cell debris was excluded from the analysis by an appropriate forward light scatter threshold setting. Compensation was used wherever necessary.

### Western blotting

At the indicated time after drug treatment, cells were collected and lysed with ice-cold lysis buffer (10 mM Tris–Cl (pH 8.0), 1 mM EDTA, 0.5 mM EGTA, 1% Triton X-100, 0.1% sodium deoxycholate, 0.1% SDS. 140 mM NaCl) for 90 min on ice. The lysates were centrifuged at 14,000 rpm for 10 min at 4 °C. Equivalent protein samples (30 μg protein extract was loaded on each lane) were subjected to SDS-PAGE gel. The proteins were then transferred onto PVDF membranes (Millipore) and blocked with 5% non-fat milk for 90 min at room temperature. The membranes, probed with the indicated primary antibodies, were incubated at 4 °C overnight. Primary antibody was detected by binding horseradish peroxidase (HRP)-conjugated anti-rabbit or anti-mouse secondary antibody with an ECL plus kit. Detection was performed using the Odyssey infrared imaging system (LI-COR, Lincoln, NE).

### Plasmids and viral transfections

pQCXIP-GFP-LC3 was constructed by inserting GFP-LC3 into BamH1 and EcoRI restriction sites of pQCXIP vector and verified by DNA sequencing. The lentiviral shRNAs human ULK1 (TRCN0000000835 and TRCN0000000836) and human AMPK (TRCN0000000857 and TRCN0000000858) were ordered from the BioMedical Genomics Center at The University of Minnesota. The negative control vector pLKO.1 has no hairpin insert, shRNA-encoding plasmids were co-transfected with envelope and packaging plasmids (VSVG, REV and pMDL) into actively growing HEK-293T cells by using the calcium phosphate transfection method. The supernatants of virus were collected 36 h after transfection, remove cell debris by centrifuging and filtering. The target cells were infected in the presence of 400 nM NVP-BEZ235. Cells were selected with 400 nM NVP-BEZ235 24 h later to generate stable cell lines and knockdown efficiency was confirmed by immunoblotting.

### Xenograft mouse model and treatment

The xenograft mouse models carrying human colon cancer HCT116 cells were established in female 5- to 6-week-old nude mice (Vital River, China), it was housed in a sterile environment with microisolator cages and allowed access to water and chow ad libitum. 1 × 10^6^ cells were resuspended in 100 μL of PBS (phosphate-buffered saline solution) and injected subcutaneously into the flanks of nude mice. Once the xenograft tumors were reaches 50–100 mm^3^, mice were treated daily with 40 mg/kg NVP-BEZ235 by oral gavage and CQ at 50 mg/kg by i.p. injection, or their combination for 7 days a week. NVP-BEZ235 was dissolved in NMP/PEG300 (1:9), sonicated in NMP firstly, and then PEG300 was added till the final volume; CQ was supplied as a stock solution. Mice terminated after 15 days treatment. Tumor growth was monitored by calipers, and tumor volumes were calculated by the formula 0.5 × length × width^2^. Mice were euthanized when tumors reached ~ 1.0 cm^3^ in size.

### Statistical analysis

Statistical analyses were carried out using the GraphPad Prism V software. All assays were repeated independently for a minimum of three times. Data are represented as mean ± SEM in the figures. p values were calculated using the Student’s paired t-test. Differences were considered statistically significant at *p < 0.05, **p < 0.01, ***p < 0.001.

## Results

### NVP-BEZ235 induced apoptosis and autophagy simultaneously

**Fig. 1:**
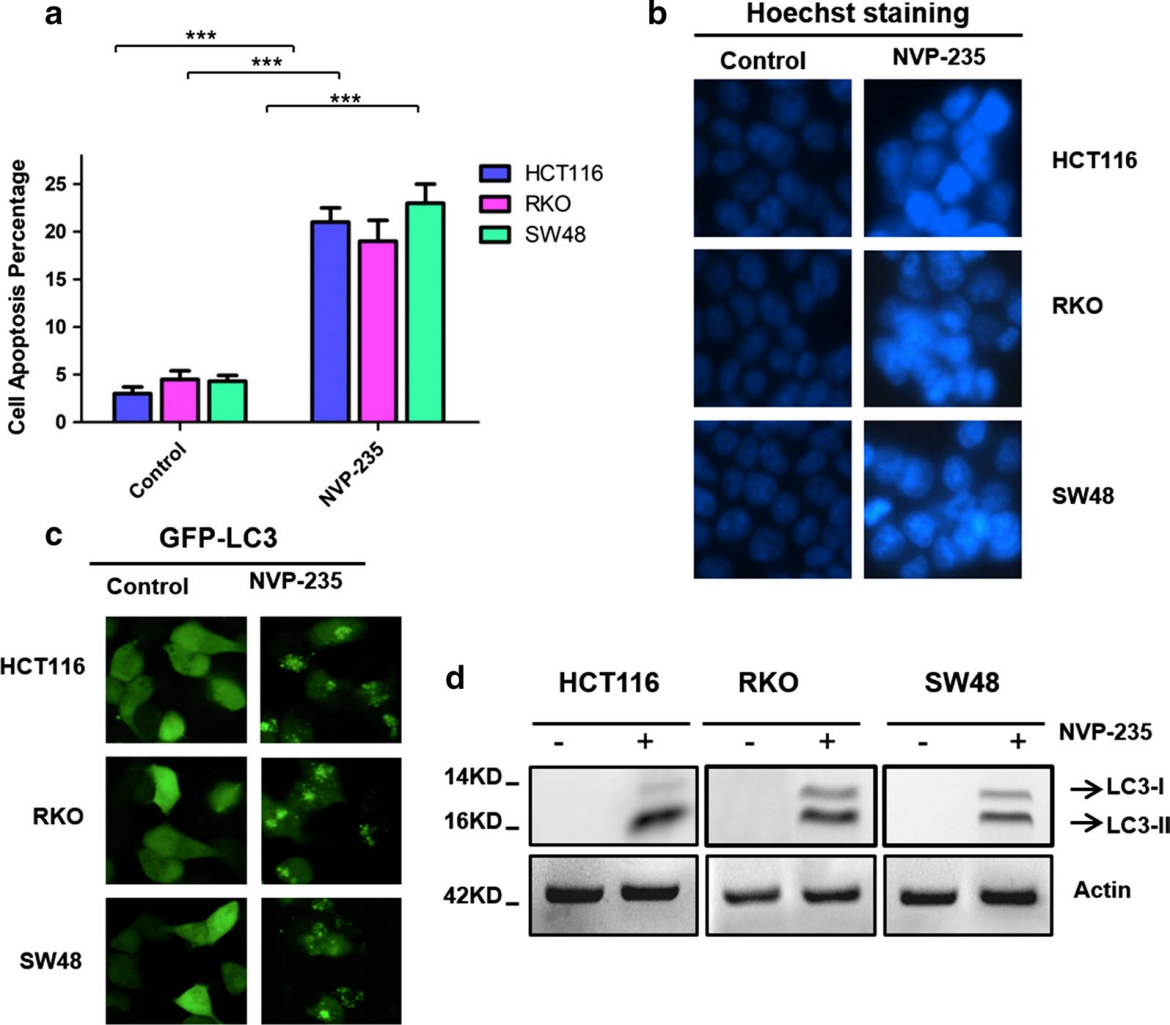
Effects of NVP-BEZ235 on apoptosis and autophagy. **a** Cell apoptosis was analyzed in various colon cancer cells using Fluorescence-activated cell sorting (FACS) technique after the treatment of 400 nM NVP-BEZ235 for 24 h. **b** Hoechst33342 morphological examination of apoptosis in HCT116, RKO, SW48 cells. Cells were treated with 400 μM NVP-BEZ235 and incubated for 24 h, then stained with hochest333342, visualized under a Nikon fluorescent microscope (60×). **c** Representative images of GFP-LC3 translocation in HCT116, RKO, SW48 cells by treated with NVP-BEZ235, GFP-LC3 puncta was visualized by fluorescence microscopy. **d** The expression of LC3-II were detected by western blotting in the presence or absence of NVP-BEZ235. Similar results were obtained from three independent experiments

NVP-BEZ235 is currently in phase1/2 clinical trials that promoted colon cancer cell apoptosis [14]. As shown in Fig. 1a, NVP-BEZ235 caused significant cell apoptosis in all analyzed colon cancer cells, including HCT116, RKO and SW48 cells. To observe cell apoptosis directly, morphological examination was performed with Hoechst33342 staining (Fig. 1b). Chromatin condensation was observed after NVP-BEZ235 treatment 24 h in HCT116, RKO and SW48 cells. In order to examine whether NVP-BEZ235 would regulate autophagy in colon cancer cells, we evaluated the puncta of GFP-LC3 in differently colon cancer cell lines by fluorescence microscope. As shown in Fig. 1c, NVP-BEZ235 treated HCT116, RKO, SW48 cells exhibited a dramatic increase in puncta formation of GFP-LC3 compared with the controls. Furthermore, we detected the LC3-II expression in three colon cancer cells to confirm the results of NVP-BEZ235 would induction of autophagy (Fig. 1d).

### NVP-BEZ235 induced autophagy through AMPK/ULK1 pathway

**Fig. 2:**
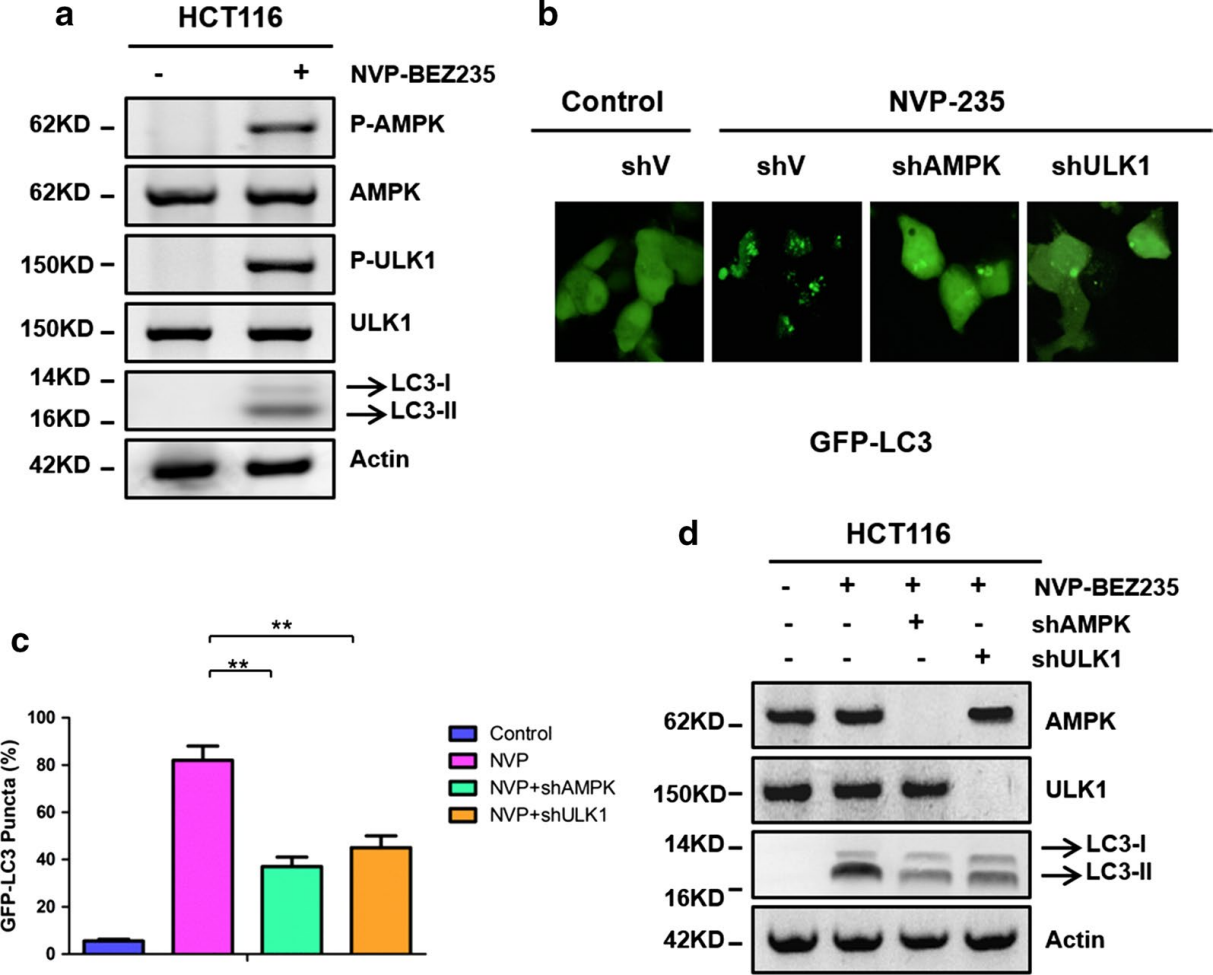
The AMPK/ULK1 axis regulated autophagy induction by NVP-BEZ235. **a** The expressions of both phosphorylated and total AMPK and ULK1, LC3-II were determined by western blotting. **b** Representative images of GFP-LC3 translocation in HCT116 cell expressing shRNAs for AMPK or ULK1 in treatment of NVP-BEZ235. **c** Quantification of GFP-LC3 translocation from the representative images shown in (**b**). **d** AMPK, ULK1 and LC3-II were detected in HCT116 cell following the treatment of NVP235, with AMPK and ULK1 knockdown or not

Many signaling pathways, including mTOR, AMPK and MAPK, which were identified to regulate autophagy [18]. We next to explore which signaling pathways play an important role in NVP-BEZ235 induced autophagy. As shown in Fig. 2a, NVP-BEZ235 increased the phosphorylation of AMPK and ULK1 expression markedly, in addition to, LC3-II, the autophagy marker, also have increased obviously. Of note, total AMPK and ULK1 expression had no change through the whole process. Lately, we detected the puncta formation of GFP-LC3 with AMPK and ULK1 depletion by shRNAs in the treatment with NVP-BEZ235 or not (Fig. 2b, c). The GFP-LC3 puncta increased markedly when treated with NVP-BEZ235 compared with the controls. However, knockdown of AMPK and ULK1 would dramatically reduce the puncta formation of GFP-LC3 in the present of NVP-BEZ235. We also found that LC3-II detected by western blotting was decreased in shAMPK or shULK1 in treat with NVP-BEZ235 (Fig. 2d). In a word, these results together suggest that NVP-BEZ235 induced autophagy through the AMPK/ULK1 pathway in colon cancer.

### Knockdown of AMPK/ULK1 inhibited cell proliferation and further promoted apoptosis

**Fig. 3:**
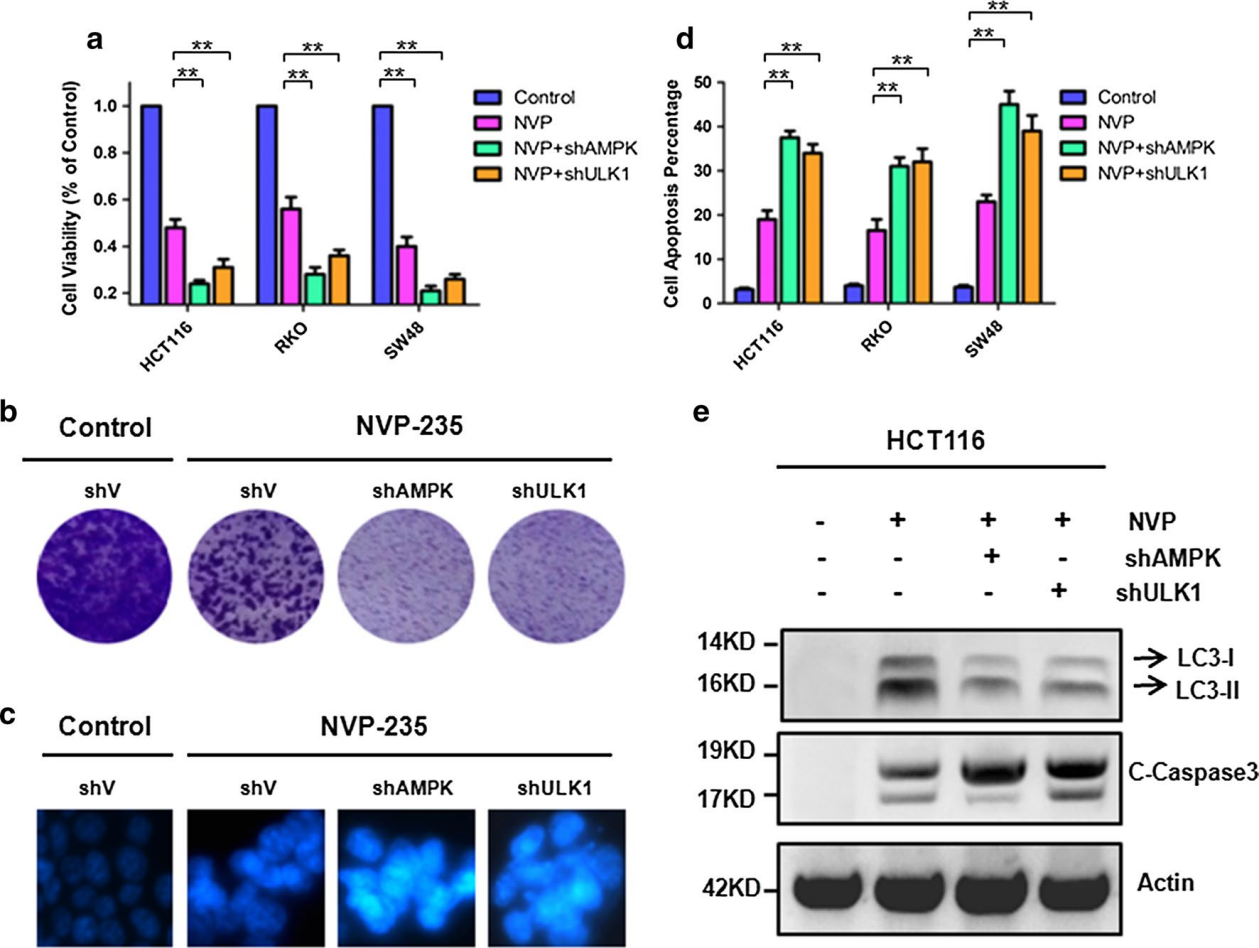
Blocking autophagy by knockdown of AMPK/ULK1 promoted apoptosis. **a** Cells viability was analyzed using Cell Counting Kit-8 after AMPK or ULK1 knockdown upon NVP-BEZ235 treatment for 24 h in the three colon cancer cells (HCT116, RKO, SW48) respectively. **b** Colony formation of shAMPK or shULK1 in the HCT116 cell, cells were treated with NVP-BEZ235, followed with crystal violet staining of attached cells at 14 days. **c** Hoechst33342 morphological examination of apoptosis in shAMPK or shULK in the HCT116 cells. Cells were treated with 400 μM NVP-BEZ235 and incubated for 24 h, then stained with hochest333342. **d** Fluorescence-activated cell sorting (FACS) assays was used to analysis cell apoptosis. Cells were treat with NVP-BEZ235 for 24 h in HCT116, RKO and SW48 with shAMPK or shULK. **e** Western blotting showing the expression of LC3-II and cleaved caspase3 in shAMPK or shULK colon cancer cell after 400 μM NVP-BEZ235 treatment

To dissect the effects of autophagy essential genes AMPK and ULK1, we measured cell viability in three colon cancer cell line. As shown in Fig. 3a, the cell viability of shAMPK or shULK1 in HCT116, RKO and SW48 cells was decreased significantly compared with that without knockdown cell lines in response to NVP-BEZ235. As shown in Fig. 3b, c, the results of crystal violet and Hoechst33342 staining also show similar effects of knocking down AMPK or ULK1 in colon cancer cells. Consistently, cell apoptosis was detected by shAMPK or shULK1 in HCT116, RKO and SW48 cells, as shown in Fig. 3d, the cell apoptosis increased after NVP-BEZ235 stimulation. Taken together, these results demonstrated that inhibit autophagy can further promote apoptosis in treat with NVP-BEZ235. Furthermore, we also detected the expression of LC3-II and C-Caspase3 in HCT116 cells (Fig. 3e). The accumulation of C-Caspase3 correlates with apoptosis activity. Our results showed that C-Caspase3 expression was increased significantly and LC3-II expression was decreased dramatically upon NVP-BEZ235 treatment in CRC cells.

### Targeting autophagy by CQ increased NVP-BEZ235 induced apoptosis

**Fig. 4:**
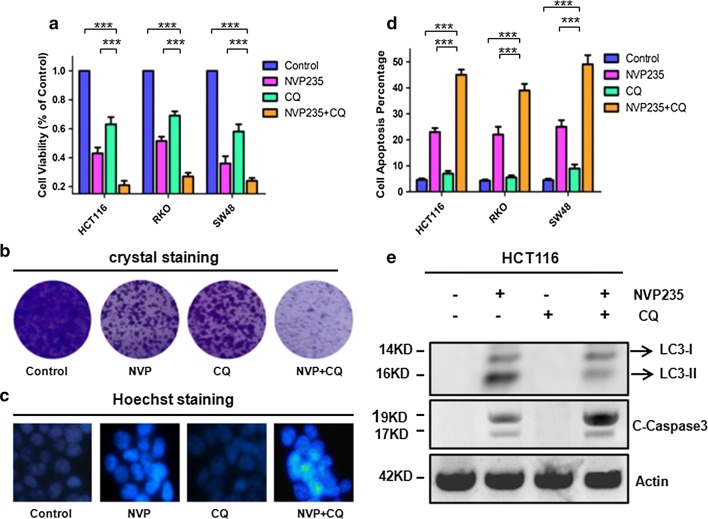
The combinational treatment of NVP-BEZ235 and CQ induces apoptosis. **a** Cell viability was analyzed by CCK-8 at NVP-BEZ235, CQ and the combination treatment of 400 nM NVP-BEZ235 and 50 μM CQ in HCT116, RKO and SW48 cells. **b** Colony formation of HCT116, RKO and SW48 cells by crystal violet staining after 24 h of different drugs treatment. **c** Hoechst33342 morphological examination of apoptosis after combination treatment for 24 h in three colon cancer cells. **d** Apoptosis in HCT-116, RKO and SW48 cells were treated with either 400 nM NVP-BEZ235, 50 μM CQ or their combination therapy was detected by flow cytometry. **e** Western blotting showing the expression of LC3-II and cleaved caspase3 after the treatment of NVP-BEZ235, CQ or their combination therapy for 24 h

The effect of NVP-BEZ235, the late stage autophagy flux inhibitor CQ, and both drugs combinational treatment was evaluated using Cell Counting Kit-8. As shown in Fig. 4a, the cell viability was decrease in treated with NVP-BEZ235 and CQ respectively, however, it is apparent that CQ does not induce apoptosis compared with NVP-BEZ235. Interesting, the combinational treatment would decrease the cell viability significantly compared to single drug treatment. Meanwhile, the similar results of the synergistic effect of their combined treatment examined by crystal violet and Hoechst33342 staining (Fig. 4b, c). Furthermore, the analogous results also were obtained from the FACS analysis, as shown in Fig. 4d. To further confirm our result, the expression of LC3-II and C-Caspase3 were detected by western blotting (Fig. 4e), their combinational treatment increase C-Caspase3 and decrease LC3-II expression evidently. These findings collectively indicated that the autophagy inhibitor CQ would promoted NVP-BEZ235 induced apoptosis. So, the combinational treatment will more effective compared to monotherapy.

### The combinational therapy of NVP-BEZ235 and CQ shows synergistic antitumor activity in colon cancer in vivo

**Fig. 5:**
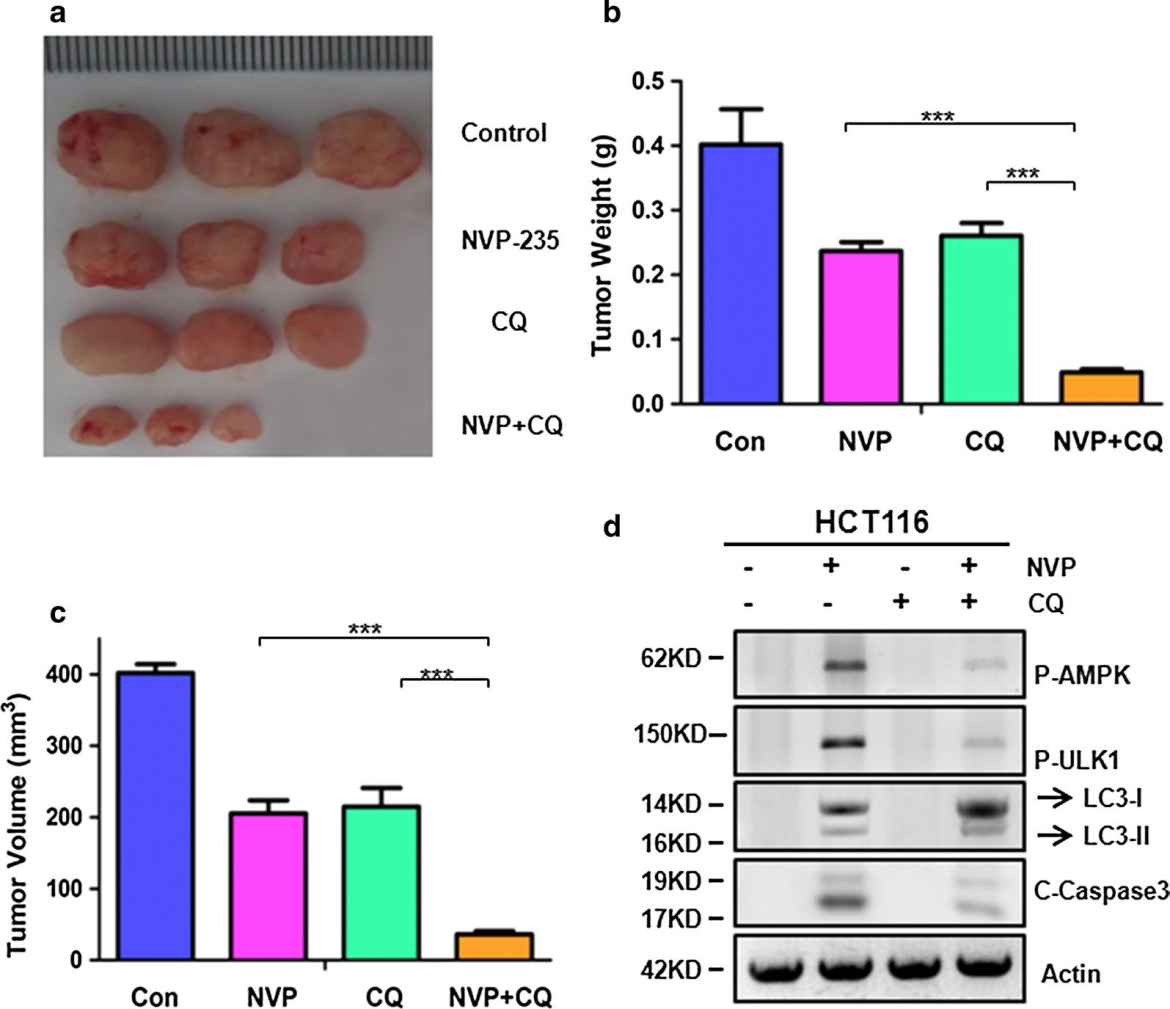
The antitumor effects of NVP-BEZ235 and CQ in vivo. **a**–**c** Nude mice were injected s.c. with 1 × 10^6^ HCT116 cells. Once the tumor was measurable, mice were treated daily with 40 mg/kg NVP-BEZ235 by oral gavage, or CQ at 25 mg/kg by i.p. injection, or their combination for 15 consecutive days. **a** Representative tumors at the end of the experiment. **b** Tumors weight. **c** Tumors volume at indicated time points after treatment was calculated. Data represent the mean ± S.D of four independent experiments. **d** p-AMPK, p-ULK1, LC3-II and cleaved-caspase3 expression were analyzed by western blotting in representative tumors. Similar results were obtained from three independent experiments

**Fig. 6:**
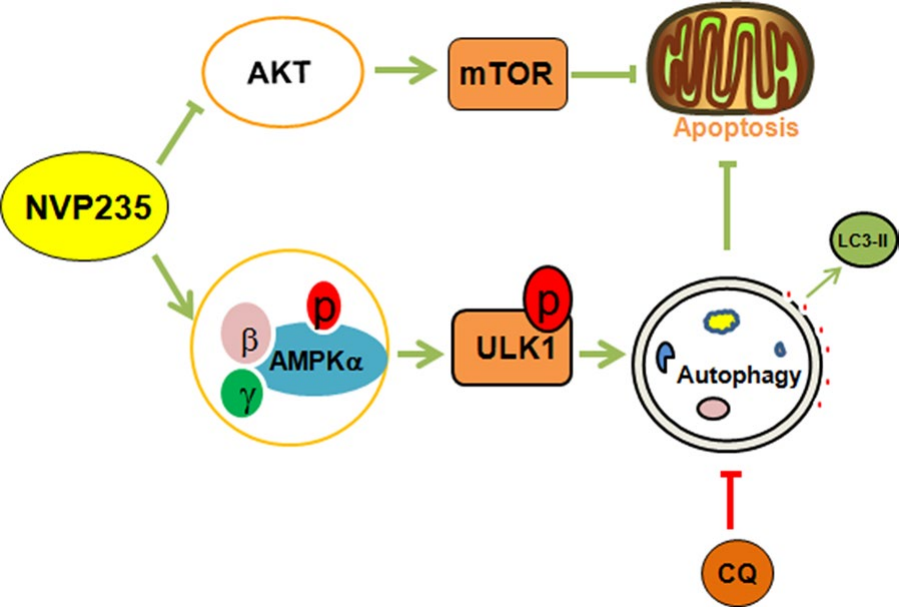
Schematic representation of the relation between apoptosis and autophagy in tumor

On the basis of our aforementioned findings, the NVP-BEZ235 and CQ combinational treatment is superior to either NVP-BEZ235 or CQ single treatment on upregulation colon cancer cells apoptosis in vitro. From this we reasoned that it would suppress human colon cancer tumor growth in vivo. In order to confirm it, we established xenograft mice model using HCT116 cells to generated subcutaneous tumors. Then the mice were divided into four groups: PBS (control), NVP-BEZ23, CQ, or NVP-BEZ235 plus CQ. As shown in Fig. 5a, the alone treat with NVP-BEZ235 or CQ showed a moderate inhibition of tumor growth, interestingly, combinational therapy with NVP-BEZ235 and CQ caused significant regression compared to monotherapy. Quantitative analysis displays that the weight and volume of tumor in combinational treatment group were less than 30% of that in NVP-BEZ235 and CQ single treatment group (Fig. 5b, c). Western blotting of p-AMPK, p-ULK1, LC3-II and C-Caspase3 expression further to confirm that results (Fig. 5d). Therefore, the combinational treatment suppressed tumor growth more effective than individual treatment in vivo. The schematic representation of the relation between apoptosis and autophagy were shown in Fig. 6.

## Discussion

Currently, the common treatment for CRC is chemotherapy and target therapy and complete surgical resection, there still no effective therapeutic options exist for CRC patients to improve survival rate and drug resistance is a serious therapeutic hurdle for them [4, 25, 26]. Therefore, novel therapeutic agents are an urgent need to improve therapeutic effectiveness. NVP-BEZ235 has been shown promising clinical activity in colon carcinoma, however, the underlying mechanisms against tumor is remain elusive. In our study, we confirmed NVP-BEZ235 can promote the initiation of autophagy and induce apoptosis. Then we found there have closely connection between autophagy and apoptosis that blocking autophagy promote NVP-BEZ235 induced apoptosis, which can increase clinical drugs sensitive to tumor therapy, indicating that target autophagy may serve as an effective strategy for colon cancer therapy. The novelty of our study resides in the demonstration that the PI3K/mTOR inhibitor NVP-BEZ235 induces autophagy by AMPK/ULK1 pathway and the combinational treatment of NVP-BEZ235 and CQ further promote apoptosis to suppress tumor growth (Fig. 6).

The activation of Akt/mTOR signaling decreased sensitivity to chemotherapy, as far as the tolerability is concerned, so we need to inhibit this pathway to increase anti-tumor drugs effect [26, 27]. Here, we have analyzed the therapeutic potential for the novel, orally available dual PI3K/mTOR inhibitor NVP-BEZ235, which has entered clinical trials with low side-effects of solid tumors, including colon cancer. We previously identified that NVP-BEZ235 induced cell apoptosis in colon cancer cell lines (Fig. 1a, b). This mechanism explains why NVP-BEZ235 exerts promising anti-cancer effects. More importantly, we have demonstrated that NVP-BEZ235 can promote autophagy through AMPK/ULK1 axis firstly (Fig. 2a–d). Indicating that AMPK/ULK1 pathway may important to anti-tumor drugs to induce autophagy for targeting cancer therapy in the future.

The autophagy inhibitor CQ is an antimalarial drug approved by the Food and Drug Administration. CQ and its derivatives (HCQ) have been administrated in our animal studies, and more which were the only autophagy inhibitors currently used for treating human tumors in clinical trials. Currently, numerous studies have indicated that using CQ as a mono or combination-therapy to blocking autophagy have a clinical benefit for antitumor, and studies have shown combination of apatinib with CQ tends to have the most significant anti-tumor effect of CRC [28]. In our vivo date, when monotherapy with CQ would inhibit colon cancer tumor growth (Fig. 5a), it is unclear whether their antitumor effects are primarily due to their ability to inhibit autophagy or have some others factor. Our study provided compelling evidence to support that NVP-BEZ235 and CQ co-treatment could be a maximum therapeutic effect for CRC in vivo (Fig. 5a). Importantly, no obvious harmful physiological consequences, monitored by body weight, are being observed with long-term administration of NVP-BEZ235 and CQ in our xenograft mice model studies. Notably, the combinational treatment provides a promising therapeutic strategy to enhance the effects of chemotherapy for colon cancer patients.

AMPK is a highly conserved Ser/Thr protein kinase complex, which is another potential candidate to regulate autophagy through maintaining energy homeostasis [29]. The molecular mechanism of AMPK regulates autophagy is generally assumed by inhibiting mTOR that acts at the initiation step of autophagy which negatively regulates autophagy by inactivating ULK1 [19, 29]. In our study, we provide molecular insights into how NVP-BEZ235 induced autophagy through AMPK/ULK1 pathway. The NVP-BEZ235 upregulation the phosphorylation expression of AMPK and ULK1 were validated our result (Fig. 2a). We also have shown that genetic inhibition of AMPK and ULK1 could significantly increase NVP-BEZ235 induced apoptosis (Fig. 3b–d). These results indicated that autophagy play a vital role in NVP-BEZ235-induced apoptosis. Additionally, a recent report investigated that blocking autophagy by silence Atg7 or Atg5 (autophagy related gene) would decreased autophagy induction after NVP-BEZ235 stimulate but not alter the induction of apoptosis which different from our results [30]. Indicating the underlying mechanism of NVP-BEZ235 induce autophagy and apoptosis are remaining elusive, we need to do further research to understand it.

The role of autophagy activation in cancer may help cancer cells to adapt intracellular environmental stress or induce cell death [16, 17]. Overwhelming preclinical and clinical evidence suggest that suppression of autophagy has been considered an opportunity to treat cancer, especially in combinational with chemotherapeutic agents [31]. Apoptosis contributed to increasing colon cancer cells sensitivity to chemotherapeutic drugs. Previous researcher demonstrated that autophagy is intimately linked with apoptosis, such as protect cells evade apoptosis. Therefore, we combination autophagy inhibitors CQ with anti-cancer drugs NVP-BEZ235 to increasing the colon cancer cells dying by enhance apoptosis sensitization (Fig. 4a–e). These findings highlight the molecular machinery of autophagy and apoptosis were significant to govern cell-fate which offer a promising insight for future clinical therapy.

In general, our study has several important implications. Firstly, for the first time, it provides novel insights into the molecular mechanisms that NVP-BEZ235 induces autophagy via an AMPK/ULK1 dependent way. Secondly, our results demonstrate that autophagy and apoptosis are antagonistic in colon cancer, the initiation of autophagy hinders apoptotic process while blocking autophagy further promotes apoptosis. Finally, targeting autophagy is a promising therapeutic option and the combinational therapy of NVP-BEZ235 and CQ shows a synergistic antitumor activity in colon cancer, which provide a novel therapeutic strategy for colon cancer.

## Conclusion

In general, our study has included several important substances: NVP-BEZ235 induce autophagy through AMPK/ULK1 pathway; Blocking autophagy by knocking down AMPK or ULK1 promote NVP-BEZ235 induced apoptosis; The combinational therapy of NVP-BEZ235 and CQ shows synergistic antitumor effects in colon cancer in vitro and in vivo.

## Data Availability

All data generated or analyzed during this study are included in this published article.

## Abbreviations

AMPK: AMP-activated protein kinase
ULK1: unc-51 like autophagy activating kinase1
mTOR: the mammalian target of rapamycin
CRC: colorectal cancer
CQ: chloroquine
